# Performance Evaluation of the VISITECT CD4 Lateral Flow Point‐Of‐Care Assay for Identifying Advanced HIV Disease

**DOI:** 10.1002/jcla.70340

**Published:** 2026-08-19

**Authors:** Mariana Andreani, Claudia E. Frola, Liliana Guelfand, Fabiana C. Chaile, José Barletta, María J. Rolón, Mariana Erbin, Juan L. Rodriguez‐Tudela

**Affiliations:** ^1^ División Laboratorio de Análisis Clínicos, Sección Microbiología Hospital General de Agudos “Dr. Juan A. Fernández” Buenos Aires Argentina; ^2^ División Infectología Hospital General de Agudos “Dr. Juan A. Fernández” Buenos Aires Argentina; ^3^ Global Action for Fungal Infections (GAFFI) Geneva Switzerland

**Keywords:** advanced HIV disease, CD4 count, diagnostic performance, lateral flow assay, point‐of‐care testing

## Abstract

**Objectives:**

To evaluate the performance and reader‐dependent variability of the VISITECT CD4 lateral flow assay (LFA) as a screening tool for identifying advanced HIV disease (AHD; CD4 ≤ 200 cells/μL) during routine programmatic implementation.

**Methods:**

We conducted a two‐phase diagnostic evaluation in Argentina, including a training phase (*n* = 100) and a validation phase (*n* = 249), for a total of 349 participants, comparing VISITECT CD4‐LFA with flow cytometry. Diagnostic performance was assessed using the manufacturer‐recommended cut‐off. Sensitivity, specificity, and accuracy were calculated with 95% confidence intervals (CI). Receiver operating characteristic (ROC) analysis was used to estimate the implicit CD4 thresholds applied by readers.

**Results:**

The VISITECT CD4‐LFA showed 100% sensitivity (95% CI 94–100) in both phases. Specificity improved from 19% (95% CI 8–35) to 40% (95% CI 31–49) after training, with accuracy increasing from 42% (95% CI 33–53) to 57% (95% CI 51–63). ROC analysis demonstrated a shift in the implicit reader threshold from 363 to 270 cells/μL, indicating improved interpretation with experience.

**Conclusions:**

The VISITECT CD4‐LFA is a highly sensitive point‐of‐care (POC) tool for AHD screening, though its visual design limits specificity, highlighting the need for reader‐independent technologies.

## Graphical abstract

The VISITECT CD4 lateral flow assay (LFA) was evaluated for identifying advanced HIV disease (CD4 ≤ 200 cells/μL) during routine programmatic implementation in Argentina. The assay showed 100% sensitivity, while specificity improved after observer training, reflecting reduced reader‐dependent variability. These findings support the use of the VISITECT CD4 as a point‐of‐care screening tool for advanced HIV disease.

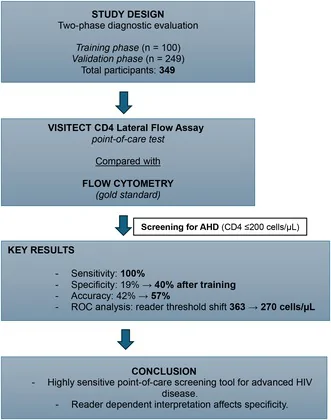

## 1 Introduction

Despite expanded access to antiretroviral therapy (ART) and the rollout of the World Health Organization's (WHO) “test‐and‐treat” strategy, the proportion of people living with HIV (PLHIV) presenting with advanced HIV disease (AHD) remains unchanged at approximately 30% [1]. This advanced disease stage, occurring both in individuals with delayed diagnosis and those re‐engaging in care, continues to pose a major challenge due to its association with high morbidity and mortality, primarily driven by opportunistic infections.

CD4 T‐cell count is the most reliable biomarker for assessing immune status, predicting disease progression risk, and guiding clinical management, including prophylaxis, screening, and treatment of opportunistic infections [2, 3, 4]. Flow cytometry is the gold standard for CD4 quantification due to its high accuracy and reproducibility. However, since flow cytometry requires centralized laboratory facilities, specialized equipment, controlled specimen transport, and trained personnel, it does not provide immediate results at the point‐of‐care (POC), which prolongs turnaround times and delays clinical decision‐making [5, 6] with potential impact on mortality. To address these challenges, a POC CD4 testing device using a lateral flow assay (LFA) format has been developed as a feasible alternative, offering a rapid and semi‐quantitative method to assess CD4 cell levels in healthcare settings without the need for specialized laboratory infrastructure [7, 8]. One such innovation is the VISITECT CD4 Advanced Disease test (VISITECT CD4‐LFA), recently developed by Accubio, which is designed to identify individuals with CD4 counts at or below 200 cells/μL according to the WHO definition of AHD.

In a previous study, we analyzed the feasibility of implementing the VISITECT CD4‐LFA as part of a package of care strategy for PLHIV with suspected AHD. The assay achieved a sensitivity of 100% for detecting CD4 counts ≤ 200 cells/μL; however, the specificity was 19% [9]. While these results highlighted the potential of the test as a screening tool due to the sensitivity score, the low specificity underscored the need for strategies to improve cost‐effective diagnostic accuracy to guide clinical decisions. Since the LFA requires visual interpretation of the intensity of one test line compared to the reference line of 200 cells/μL, its performance may be significantly affected by the operator's training, experience, visual acuity, and innate ability, making it a challenging variable to control when the test is deployed globally in a public health approach.

This study evaluated the diagnostic performance of the VISITECT CD4‐LFA under routine implementation conditions and explored the impact of visual interpretation and accumulated reader experience on specificity and operational classification thresholds.

The 2021 cohort corresponds to data previously reported as part of a package‐of‐care implementation study [9]. In the present analysis, this cohort was considered the initial implementation phase. The 2023–2025 cohort was enrolled during a subsequent prospective implementation phase in which the VISITECT CD4‐LFA continued to be used for rapid identification of advanced HIV disease. The two phases were compared to evaluate test performance during routine use and to assess changes in reader‐dependent variability over time.

The aim of this study is to evaluate the diagnostic performance and reader‐dependent variability of the VISITECT CD4‐LFA compared with flow cytometry for identifying AHD (CD4 ≤ 200 cells/μL) during routine programmatic implementation. Specifically, this analysis aimed to determine whether the visually interpreted LFA systematically overclassified patients with AHD in the training period, and to identify its operational threshold under real‐use conditions.

## 2 Methods

### 2.1 Study Design

This was a two‐phase diagnostic evaluation carried out as part of an implementation project to enhance rapid diagnosis of opportunistic infections in PLHIV using POC tests.

### 2.2 Study Population

Participants were consecutively enrolled and included adults aged ≥ 18 years with documented HIV infection, including individuals with newly diagnosed HIV infection, those with known HIV infection, and those re‐engaging in HIV care.

Individuals with an undetectable viral load within the three months prior to enrollment were excluded.

### 2.3 Study Procedures

The first phase *(training)* was conducted between June and October 2021 at Hospital Juan A. Fernández in Buenos Aires, Argentina, and corresponds to a previously published cohort. During this phase, a diagnostic performance evaluation was performed using 100 EDTA whole blood samples collected from PLHIV, which were analyzed in parallel using the VISITECT CD4‐LFA and flow cytometry (FACSCalibur, Becton Dickinson).

The second phase (*validation*) was carried out between November 30, 2023, and August 29, 2025. In this phase, CD4 counts were evaluated in 249 EDTA whole blood samples from PLHIV using both the VISITECT CD4‐LFA test and flow cytometry.

### 2.4 Training

Prior to the first phase, healthcare personnel received formal training on the use of the VISITECT CD4‐LFA test, followed by a refresher training session before the second phase. To support capacity building, AccuBio provided structured training materials, customized virtual workshops, and access to the Global Learning Center (GLC) e‐learning platform. The training covered theoretical foundations, practical demonstration of the VISITECT CD4‐LFA procedure, guidance on critical steps (such as timing and interpretation), and interactive Q&A to resolve doubts in real time. The training was user‐focused and competency‐based, designed to ensure high‐quality implementation and minimize inter‐operator variability.

### 2.5 Test Procedure

VISITECT CD4‐LFA testing was performed using EDTA whole blood samples according to the manufacturer's instructions. Results were interpreted visually by trained laboratory biochemists and technicians by comparing the intensity of the test band with the reference band corresponding to 200 cells/μL. All tests were performed in a laboratory setting.

The two study phases were conducted sequentially, allowing assessment of test performance over time as operators became progressively familiar with the VISITECT CD4‐LFA during routine use.

Three operators performed the test consistently throughout both study phases. During the validation phase, one additional operator joined the team after receiving the same structured training.

Flow cytometry (FACSCalibur, Becton Dickinson) was used as the reference standard for CD4 count determination.

Statistical analyses were conducted using R (RStudio version 2025.05.1). The diagnostic performance of the VISITECT CD4‐LFA, including sensitivity, specificity, accuracy, and positive and negative predictive values, was evaluated for both phases using the manufacturer‐recommended cut‐off.

ROC curve analysis was performed to determine the implicit CD4 thresholds at which readers classified patients as having AHD or non–AHD using the VISITECT CD4‐LFA. In this analysis, the binary variable represented the observer's visual interpretation of the LFA result according to the manufacturer's instructions, whereas the continuous variable (CD4 count measured by flow cytometry) served as the reference standard. The ROC curve did not evaluate the diagnostic performance of the LFA itself but instead identified the CD4 level corresponding to the decision boundary implicitly applied by observers when interpreting band intensity. Training, validation, and combined datasets were analyzed separately.

### 2.6 Ethics

The study protocol was approved by the local Institutional Review Board under registry numbers 3370 (November 9, 2020) and 8788 (March 3, 2023). Written informed consent was obtained from all participants. The study was conducted in accordance with Good Clinical Practice and Good Laboratory Clinical Practice guidelines, as well as all applicable local and international regulations.

## 3 Results

The diagnostic performance of the VISITECT CD4‐LFA was evaluated in our laboratory in two phases, including a total of 349 participants (*Training phase: n* = 100*; Validation phase: n* = 249). The median age of participants was 39 years (IQR 32–48).

Among the 349 individuals, VISITECT CD4‐LFA classified 297 (85%) with CD4 ≤ 200 cells/μL, while 199 (57%) had this CD4 count confirmed by flow cytometry. Among patients with CD4 > 200 cells/μL by flow cytometry, 65.3% (98 of 150) were misclassified as having CD4 ≤ 200 cells/μL.

The median CD4 count among individuals classified as ≤ 200 cells/μL by VISITECT CD4‐LFA was 124 cells/μL (IQR 49–192), compared with 440 cells/μL (IQR 369–1104) among those classified as > 200 cells/μL.

The raw 2 × 2 contingency tables used to calculate diagnostic performance for the training, validation, and combined datasets are presented in Table 1.

**TABLE 1:** VISITECT CD4‐LFA versus flow cytometry: Training (2‐a), validation (2‐b), and combined (2‐c) performance using the manufacturer's cut‐off of ≤ 200 cells/μL.

1‐a Training phase			
	Flow Cytometry		
		≤ 200 CD4	> 200 CD4
VISITECT CD4‐LFA	≤ 200 CD4	63	30
	> 200 CD4	0	7

1‐b Validation phase			
	Flow Cytometry		
		≤ 200 CD4	> 200 CD4
VISITECT CD4‐ LFA	≤ 200 CD4	136	68
	> 200 CD4	0	45

1‐c Combined			
	Flow Cytometry		
		≤ 200 CD4	> 200 CD4
VISITECT CD4‐LFA	≤ 200 CD4	199	98
	> 200 CD4	0	52

Sensitivity for the detection of a CD4 count ≤ 200 cells/μL was 100% (95% CI: 94–100) in both phases, whereas specificity increased from 19% (95% CI: 8–35) in the training phase to 40% (95% CI: 31–49) in the validation phase. Accuracy also improved from 42% (95% CI: 33–53) to 57% (95% CI: 51–63).

Considering the estimated prevalence of CD4 ≤ 200 cells/μL among individuals with new HIV diagnosis in Argentina (28.8%) [10], the positive predictive value was 33% in the training phase and 40% in the validation phase, while the negative predictive value was 100% in both study periods (Table 2).

**TABLE 2:** Diagnostic performance of the VISITECT CD4‐LFA compared with flow cytometry.

*Performance Metric*	VISITECT *CD4–LFA*		
	*Training phase (n = 100)*	*Validation phase (n = 249)*	*Combined (n = 349)*
Sensitivity, % (95% CI)	100 (94–100)	100 (97–100)	100 (98–100)
Specificity, % (95% CI)	19 (8–35)	40 (31–49)	35 (27–43)
Accuracy, % (95% CI)^a^	42 (33–53)	57 (51–63)	53 (48–59)
Positive Predictive value, % (95% CI)^a^	33 (30–37)	40 (37–44)	38 (35–41)
Negative Predictive value, % (95% CI)^a^	100 (59–100)	100 (92–100)	100 (93–100)

^a^ Values adjusted for the prevalence of advanced HIV disease in Argentina (28.8%) [10].

Based on the observed diagnostic performance (Table 2), CD4 counts obtained by flow cytometry were compared with the binary VISITECT CD4‐LFA classifications (CD4 ≤ 200 cells/μL vs. CD4 > 200 cells/μL) through receiver operating characteristic (ROC) curve analysis to identify the implicit CD4 thresholds applied by readers during visual interpretation. In the training dataset, the ROC AUC was 96.0% (95% CI 61.5–98.1); in the validation dataset, 95.2% (92.0–97.3); and in the combined dataset, 95.7% (93.1–97.4). The ROC–derived thresholds corresponding to reader interpretation were 363 cells/μL in training and 270 cells/μL in validation and overall, indicating improved reader discrimination of band‐intensity differences with experience.

## 4 Discussion

This study evaluated the diagnostic performance of the VISITECT CD4‐LFA for identifying CD4 ≤ 200 cells/μL among PLHIV with suspected AHD across two consecutive phases reflecting increasing operator experience with the VISITECT CD4‐LFA. The assay identified all patients with CD4 ≤ 200 cells/μL, achieving 100% sensitivity, but showed limited specificity (Table 2). A total of 98 participants (65.3% of those with a flow cytometry CD4 count > 200 cells/μL) were misclassified as having the condition, indicating that while the test effectively detects patients with low CD4 counts, it tends to overclassify those with higher values as having low CD4 counts.

The sensitivity observed in our study was in line with previous evaluations of the VISITECT CD4‐LFA, all of which have reported values above 90% for identifying CD4 ≤ 200 cells/μL [8, 11, 12, 13]. In contrast, specificity was lower than in other studies. However, recent implementation studies conducted under routine‐use conditions have also reported reduced specificity during real‐world deployment [14]. This may be partly explained by the distribution of false‐positive results, which were concentrated near the clinical decision threshold. Among the 98 false positives, the median CD4 count was 254 cells/μL (IQR: 244–322), similar to that reported by Ndlovu et al. [11] (252 cells/μL; IQR: 222–306). These findings suggest that misclassification mainly occurs in samples with CD4 values close to 200 cells/μL, where subtle visual differences are difficult to interpret, contributing to reduced specificity.

The ROC approach, although unconventional, provided a quantitative estimate of the visual threshold applied by readers, rather than a measure of diagnostic accuracy per se. Our ROC analysis showed that this behavior seems to be primarily influenced by the visual design of the test, which requires subjective comparison between two bands. When readers are aware that the assay is intended to detect severe immunosuppression, an unconscious bias may arise toward classifying results as CD4 ≤ 200 cells/μL. In particular, bands that appear slightly more intense than the reference line are difficult to interpret and may lead readers to adopt a cautious approach prioritizing sensitivity over specificity.

Training and accumulated experience, as observed between the training and validation phases, partially corrected this bias, reflected by the downward shift in the implicit decision threshold from 363 cells/μL to 270 cells/μL. This shift illustrates a learning effect, whereby experienced readers better discriminate subtle differences in band intensity. However, full standardization remains unattainable: hundreds of observers with different visual acuity, training, experience, and testing conditions make consistent interpretation virtually impossible in most real‐world settings. Consequently, specificity is unlikely to improve beyond the modest levels observed in this study, limiting the test's cost‐effectiveness in settings with lower prevalence of AHD and potentially in low‐ and middle‐income countries.

From a clinical standpoint, these findings should not be interpreted as supporting expansion of screening criteria beyond established CD4 thresholds. CD4 thresholds are useful public‐health tools, but they are not absolute biological boundaries for infectious disease risk. Current WHO guidelines continue to recommend CD4‐based screening strategies for advanced HIV disease and cryptococcal disease prevention among people living with HIV [3, 15]. Misclassification was mainly observed in individuals with CD4 counts close to 200 cells/μL, reflecting a tendency of the assay to overclassify borderline results as ≤ 200 cells/μL. The clinical relevance of this finding is pathogen specific. Tuberculosis evaluation in people living with HIV is not restricted to CD4 counts ≤ 200 cells/μL. Histoplasmosis also differs from cryptococcosis: *Histoplasma* spp. is a primary fungal pathogen, although advanced HIV disease markedly increases the risk of severe and disseminated disease. Therefore, our findings do not support modifying current screening thresholds, but they do show that CD4‐based risk stratification should be interpreted within the appropriate clinical and epidemiological context. The low specificity of VISITECT CD4‐LFA limits its clinical and economic value and reinforces the need for standardized, reader‐independent point‐of‐care CD4 technologies.

Results may differ in other settings due to uncontrolled factors such as training level, visual ability, and local quality control. Each center implementing the VISITECT CD4‐LFA should perform its own validation and maintain continuous internal quality control to ensure reliability. Future work will include a multicenter evaluation to validate these findings across settings with different levels of operator experience.

This study has some limitations. The sample size may have affected the precision of the estimates, particularly for specificity, as reflected by the relatively wide confidence intervals. Additionally, many false‐positive results clustered near the clinical decision threshold, further contributing to classification uncertainty. The single‐site design and the use of trained laboratory personnel may limit generalizability to other settings, especially decentralized or point‐of‐care contexts. Inter‐reader variability and batch‐to‐batch variation were not formally assessed and may have influenced performance.

Early identification and prompt management of AHD remain crucial to reduce early mortality, as most deaths occur within the first weeks of care due to delayed diagnosis and undetected opportunistic infections. This challenge is particularly evident in Latin America, where in Guatemala more than half of deaths among newly diagnosed HIV patients occurred within 30 days, and in Paraguay, 30‐day mortality reached 16% among those with opportunistic infections compared to 7% among those without, with histoplasmosis showing the highest fatality rates [16, 17]. Achieving automation of CD4 estimation at the POC is not merely a technical improvement but an equity imperative: it would ensure timely, standardized risk assessment for PLHIV in all resource settings.

In conclusion, the VISITECT CD4‐LFA is a highly sensitive POC tool for rapidly identifying patients with advanced HIV disease in line with WHO guidelines. However, its visual design inherently limits specificity. Automation through digital or AI‐assisted semi‐quantitative reading—similar to the CrAgSQ test (IMMY, OK, USA)—is essential to reduce observer‐dependent variability, improve reproducibility, and strengthen both the clinical and economic value of this promising assay [18].

## Funding

The implementation study was funded by the Centers for Disease Control and Prevention (CDC) through grant NU51CK000319, and by the Global Action For Fungal Infections (GAFFI), Geneva, Switzerland. CDC and GAFFI did not have access to patient‐level data.

## Ethics Statement

The study protocol was approved by the local Institutional Review Board (IRB) under registry numbers: 3370 (November 9, 2020) and 8788 (March 3, 2023).

## Consent

Written informed consent was obtained from all participants.

## Conflicts of Interest

The authors declare no conflicts of interest.

## Data Availability

The data that support the findings of this study are available from the corresponding author upon reasonable request.

## References

[1] A. Lehman , J. Ellis , E. Nalintya , N. C. Bahr , A. Loyse , and R. Rajasingham , “Advanced HIV Disease: A Review of Diagnostic and Prophylactic Strategies,” HIV Medicine 24, no. 8 (2023): 859–876, 10.1111/hiv.13487. 37041113 PMC10642371

[2] E. Zaniewski , C. H. Dao Ostinelli , F. Chammartin , et al., “Trends in CD4 and Viral Load Testing 2005 to 2018: Multi‐Cohort Study of People Living With HIV in Southern Africa,” Journal of the International AIDS Society 23, no. 7 (2020): e25546, 10.1002/jia2.25546. 32640106 PMC7343336

[3] World Health Organization , Guidelines for Managing Advanced HIV Disease and Rapid Initiation of Antiretroviral Therapy (WHO, 2017). 29341560

[4] N. Ford , G. Meintjes , A. Calmy , et al., “Managing Advanced HIV Disease in a Public Health Approach,” Clinical Infectious Diseases 66, no. Suppl 2 (2018): S106–S110, 10.1093/cid/cix1139. 29514232 PMC5850613

[5] D. K. Glencross , L. M. Coetzee , M. Faal , et al., “Performance Evaluation of the Pima CD4 Point‐Of‐Care Device in Managing Antiretroviral Therapy in South Africa,” Journal of Acquired Immune Deficiency Syndromes 61, no. 1 (2012): e8–e13, 10.1097/QAI.0b013e31826f9e3b.22918129

[6] W. Stevens , N. Gous , N. Ford , and L. E. Scott , “Feasibility of HIV Point‐Of‐Care Tests for Resource‐Limited Settings: Challenges and Solutions,” BMC Medicine 12 (2014): 173, 10.1186/s12916-014-0173-7. 25197773 PMC4157150

[7] P. K. Drain and C. Rousseau , “Point‐Of‐Care Diagnostics: Extending the Laboratory Network to Reach the Last Mile,” Current Opinion in HIV and AIDS 12, no. 2 (2017): 175–181, 10.1097/COH.0000000000000341. 28079591 PMC5287256

[8] T. Gils , J. Hella , B. K. M. Jacobs , et al., “A Prospective Evaluation of the Diagnostic Accuracy of the Point‐Of‐Care VISITECT CD4 Advanced Disease Test in Seven Countries,” Journal of Infectious Diseases 231, no. 1 (2025): e82–e90, 10.1093/infdis/jiae374. 39046150 PMC11793025

[9] M. Andreani , C. E. Frola , D. H. Cáceres , et al., “Rapid CD4 Cell Count Determination and Cryptococcus and Histoplasma Antigen Detection in People Living With HIV: Implementation of a Package of Care Strategy in a Pilot Study, Argentina,” IJID Regions 12 (2024): 100403, 10.1016/j.ijregi.2024.100403. 39188885 PMC11345679

[10] Dirección de Respuesta al VIH, ITS, Hepatitis Virales y Tuberculosis , Boletín N° 40: Respuesta al VIH y las ITS en la Argentina (Ministerio de Salud de la Nación, 2023).

[11] Z. Ndlovu , L. Massaquoi , N. E. Bangwen , et al., “Diagnostic Performance and Usability of the VISITECT CD4 Semi‐Quantitative Test for Advanced HIV Disease Screening,” PLoS One 15, no. 4 (2020): e0230453, 10.1371/journal.pone.0230453. 32243435 PMC7122771

[12] K. Lechiile , T. B. Leeme , M. W. Tenforde , et al., “Laboratory Evaluation of the VISITECT Advanced Disease Semiquantitative Point‐Of‐Care CD4 Test,” Journal of Acquired Immune Deficiency Syndromes 91, no. 5 (2022): 502–507, 10.1097/QAI.0000000000003072. 36084198 PMC9646408

[13] E. Nalintya , P. Sekar , O. L. Namakula , et al., “The Diagnostic Performance of the Visitect Advanced Disease Point‐Of‐Care CD4 Platform: A Pragmatic Mixed‐Methods Multisite Validation, Costing, and Qualitative Analysis,” Journal of Acquired Immune Deficiency Syndromes 97 (2024): 387–396, 10.1097/QAI.0000000000003505. 39159398 PMC11732718

[14] P. de Haas , P. Hepple , Y. Babo , et al., “VISITECT CD4 Advanced Disease Assay in Routine Use: Diagnostic Accuracy and Usability, Ethiopia and Indonesia,” Tropical Medicine & International Health 30, no. 7 (2025): 685–693, 10.1111/tmi.14124. 40387386 PMC12213310

[15] World Health Organization , Guidelines for Diagnosing, Preventing and Managing Cryptococcal Disease Among Adults, Adolescents and Children Living With HIV (WHO, 2022). 35797432

[16] N. Medina , A. Alastruey‐Izquierdo , O. Bonilla , et al., “A Rapid Screening Program for Histoplasmosis, Tuberculosis, and Cryptococcosis Reduces Mortality in HIV Patients From Guatemala,” Journal of Fungi 7, no. 4 (2021): 268, 10.3390/jof7040268. 33916153 PMC8065950

[17] G. Aguilar , G. Lopez , O. Sued , et al., “Implementation of a Rapid Diagnostic Assay Package for Cryptococcosis, Histoplasmosis and Tuberculosis in People Living With HIV in Paraguay,” BMC Infectious Diseases 24, no. 1 (2024): 406, 10.1186/s12879-024-09257-5. 38627642 PMC11020460

[18] D. Bermejo‐Peláez , A. Alastruey‐Izquierdo , N. Medina , et al., “Artificial Intelligence‐Driven Mobile Interpretation of a Semi‐Quantitative Cryptococcal Antigen Lateral Flow Assay,” IMA Fungus 15, no. 1 (2024): 27, 10.1186/s43008-024-00158-5. 39215368 PMC11365246

